# Revisiting and Updating the Interaction between Human Serum Albumin and the Non-Steroidal Anti-Inflammatory Drugs Ketoprofen and Ketorolac

**DOI:** 10.3390/molecules29133001

**Published:** 2024-06-24

**Authors:** Rita S. Cunha, Pedro F. Cruz, Telma Costa, Zaida L. Almeida, Marco Edilson Freire de Lima, Carlos Serpa, Otávio A. Chaves

**Affiliations:** 1 Department of Chemistry, Coimbra Chemistry Centre-Institute of Molecular Sciences (CQC-IMS), University of Coimbra, Rua Larga, 3004-535 Coimbra, Portugal; 2 Departament of Organic Chemistry, Institute of Chemistry, Federal Rural University of Rio de Janeiro, Seropédica 23890-000, RJ, Brazil

**Keywords:** non-steroidal anti-inflammatory drugs, spectroscopy, in silico calculations, biophysical characterization, pharmacokinetics

## Abstract

Ketoprofen (KTF) and ketorolac (KTL) are among the most primarily used non-steroidal anti-inflammatory drugs (NSAIDs) in humans to alleviate moderate pain and to treat inflammation. Their binding affinity with albumin (the main globular protein responsible for the biodistribution of drugs in the bloodstream) was previously determined by spectroscopy without considering some conventional pitfalls. Thus, the present work updates the biophysical characterization of the interactions of HSA:KTF and HSA:KTL by ^1^H saturation-transfer difference nuclear magnetic resonance (^1^H STD-NMR), ultraviolet (UV) absorption, circular dichroism (CD), steady-state, and time-resolved fluorescence spectroscopies combined with in silico calculations. The binding of HSA:NSAIDs is spontaneous, endothermic, and entropically driven, leading to a conformational rearrangement of HSA with a slight decrease in the α-helix content (7.1% to 7.6%). The predominance of the static quenching mechanism (ground-state association) was identified. Thus, both Stern–Volmer quenching constant (*K_SV_*) and binding constant (*K_b_*) values enabled the determination of the binding affinity. In this sense, the *K_SV_* and *K_b_* values were found in the order of 10^4^ M^−1^ at human body temperature, indicating moderate binding affinity with differences in the range of 0.7- and 3.4-fold between KTF and KTL, which agree with the previously reported experimental pharmacokinetic profile. According to ^1^H STD-NMR data combined with in silico calculations, the aromatic groups in relation to the aliphatic moiety of the drugs interact preferentially with HSA into subdomain IIIA (site II) and are stabilized by interactions via hydrogen bonding and hydrophobic forces. In general, the data obtained in this study have been revised and updated in comparison to those previously reported by other authors who did not account for inner filter corrections, spectral backgrounds, or the identification of the primary mathematical approach for determining the binding affinity of HSA:KTF and HSA:KTL.

## 1. Introduction

Non-steroidal anti-inflammatory drugs (NSAIDs) constitute a class of widely used medications for pain management and inflammation control. Their popularity stems from their efficacy in managing various conditions, ranging from mild to moderate discomfort, caused by, e.g., headaches, menstrual cramps, and arthritis. Unlike steroidal anti-inflammatory drugs, NSAIDs achieve their effects through the inhibition of the enzyme cyclooxygenase (COX), thereby impeding the production of prostaglandins, which are key mediators of inflammation and pain [1,2]. More than 30 million people worldwide use NSAIDs every day. Annually, over 70 million prescriptions are written in the United States of America (USA), with 60% originating from the over-the-counter analgesic market. Additionally, nearly 10 million prescriptions are issued in Canada, and roughly 20 million are issued in the United Kingdom (UK) [3]. In Europe, the annual dispensation of NSAIDs increases each year, with the most NSAID prescriptions being ibuprofen (IBU), diclofenac, acetylsalicylic acid, nimesulide, ketorolac, ketoprofen, glucosamine, and meloxicam [4,5,6].

NSAIDs, being primarily administered orally, enter the bloodstream through the gastrointestinal tract and then circulate throughout the body [1]. Human serum albumin (HSA), the main globular protein in the human bloodstream, plays many important roles including maintaining the intravascular colloid osmotic pressure, neutralizing toxins, preventing the photodegradation of folic acid, exhibiting neuroprotective and recovery enhancement effects, maintaining blood–brain barrier (BBB) integrity, and reducing neuronal oxidative stress and apoptosis, and it is the main carrier of various substances, including fatty acids (FAs), bilirubin, metal ions, hormones, and NSAIDs [7,8,9,10,11,12,13,14,15]. The binding of NSAIDs to albumin can impact their unbound concentration in the blood (the fraction of the drug responsible for exerting pharmacological effects), affecting the residence time, efficacy, safety, distribution, metabolism, and elimination of NSAIDs in the body, i.e., influencing their pharmacokinetics and pharmacodynamics properties [7,16]. A highly relevant aspect regarding the use of NSAIDs is the possibility of the emergence of acute kidney injury (AKI) in patients exposed to long-term treatments, which justifies the need for more refined studies on the pharmacokinetics of these medications [17]. Recently, Li and coworkers [18] reported HSA-based nanoparticles (HSNs) as potential nanocarriers for the long-term treatment of diseases; e.g., the co-encapsulation of methotrexate and glycyrrhizic acid with HSN improved their residence time to achieve anti-inflammatory efficacy and reduced hepatotoxicity.

The HSA structure (585 amino acid residues) comprises three homologous domains (I, II, and III), which are arranged into two subdomains (A and B) [19,20,21]. Sudlow and coworkers [22] were one of the first researchers to characterize the specific binding sites of different drugs, including IBU. In this case, they identified the subdomain IIA (site II) as the main binding site for IBU, which was subsequently confirmed by X-ray crystallographic data [23], and for this reason, this NSAID is widely used as a site marker in drug-displacement assays with albumin [24,25]. Recently, there have been different reports about the determination of the thermodynamic parameters and binding affinity between albumin and NSAIDs by multiple spectroscopic techniques with different mathematical approaches, e.g., double logarithmic approximation, the Stern–Volmer equation, the modified Stern–Volmer equation, and the Klotz model [26,27,28,29,30,31,32]. Unfortunately, most approaches do not consider inner filter correction in the steady-state fluorescence data and do not recognize the main fluorescence quenching mechanism using reliable instrumental methods. This step is essential for determining the best mathematical approximation to be used for binding affinity, which also influences the thermodynamic approach.

Ketoprofen (KTF) and ketorolac (KTL) are among the most used NSAIDs in humans to alleviate moderate pain and to treat some diseases, e.g., rheumatoid arthritis, osteo-arthritis, and dysmenorrhea [33,34,35,36]. However, their binding affinity with albumin was primarily determined by spectroscopy without considering the pitfalls raised above [28,29,30,31]. Thus, the present work aims to update the determination of the binding affinities of HSA:KTF and HSA:KTL by using the ^1^H saturation-transfer difference nuclear magnetic resonance (^1^H STD-NMR), ultraviolet (UV) absorption, circular dichroism (CD), steady-state, and time-resolved fluorescence techniques combined with in silico calculations. These multi-experimental methodologies were critical in identifying a feasible mathematical approach to determine the quantitative binding parameters [37] and correct those reported in the literature.

## 2. Results

### 2.1. A Qualitative Evaluation on the Binding of HSA:NSAIDs

**Figure 1:**
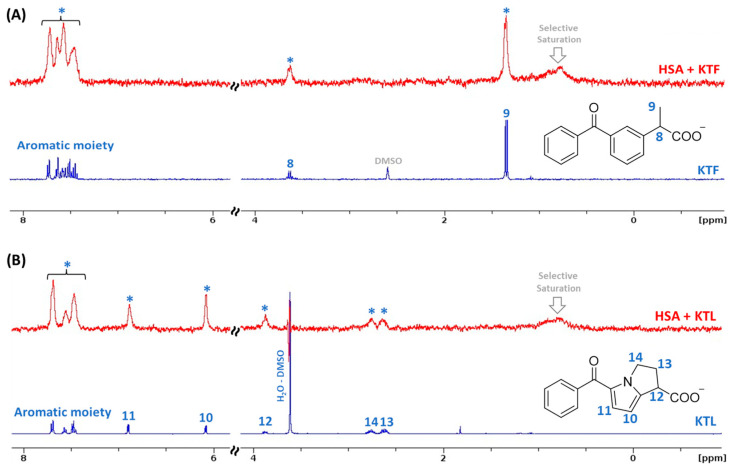
The ^1^H NMR spectrum of free (**A**) KTF and (**B**) KTL (spectra in blue). The ^1^H STD-NMR spectrum of a mixture containing HSA (20 × 10^−6^ M) in the presence of (**A**) KTF or (**B**) KTL (400 × 10^−6^ M) at pH 7.4 in D_2_O (spectra in red). The asterisk (*) represents the protein–drug interaction signals.

The ^1^H STD-NMR method is a simple, fast, and robust method focused on the signals of the ligand without any need to process NMR information about the receptor to the study of protein–ligand interactions [38,39]. Additionally, ^1^H STD-NMR has been used as a powerful tool to validate, even in parts, in silico results through the structure–binding affinity relationship [40,41]. In this sense, the preliminary binding capacity of NSAIDs to albumin was evaluated by ^1^H STD-NMR. Figure 1 depicts the ^1^H NMR signals of KTF and KTL without albumin (in blue) in a phosphate-buffered solution (PBS, pH 7.4). In this case, all ^1^H NMR signals are in accordance with the chemical structures of the NSAIDs under study [42], e.g., aromatic moieties in the 7–8 ppm range for both drugs and a multiplet around 3.5 ppm corresponding to the hydrogen closest to the carboxylic group for KTF. KTL followed the same trend identified for KTF. The ^1^H STD-NMR spectra for each NSAID are also shown in Figure 1 (in red). All ^1^H signals for KTF and KTL show STD signals, proving the binding of the ligands to the protein. In addition, signal broadening was evidenced, indicating that the NSAIDs are buried into the protein’s cavity and the binding affinity is not weak.

The saturation transfer efficiency is quantitatively expressed by the amplification factor (A_STD_, the average number of transient contacts of the ligand per molecule of receptor within a given saturation time) [43,44]. However, the significant signal broadening of NSAIDs after binding to albumin does not enable the determination of the binding epitope of each ligand [45].

**Figure 2:**
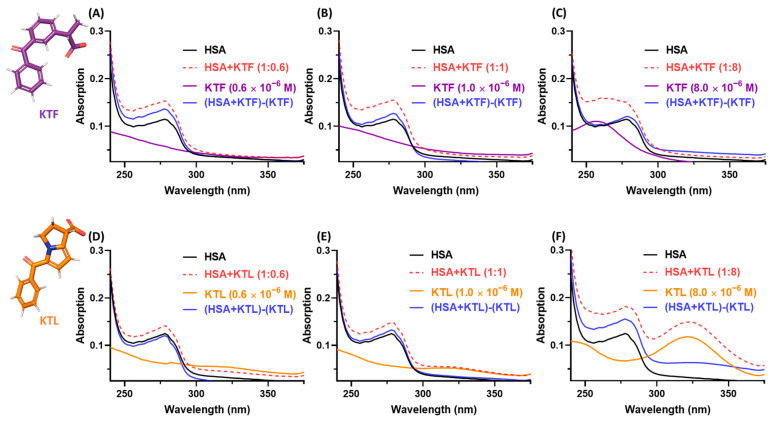
The absorption spectra for albumin (1.0 × 10^−6^ M) with or without KTF in the molar ratios of (**A**) 1:0.6, (**B**) 1:1, and (**C**) 1:8 at 310 K and pH 7.4. The absorption spectra for albumin (1.0 × 10^−6^ M) with or without KTL in the molar ratios of (**D**) 1:0.6, (**E**) 1:1, and (**F**) 1:8 at 310 K and pH 7.4. The concentrations of NSAIDs used are indicated in each spectrum.

Combining the described ^1^H STD-NMR data above with the UV absorption analysis offers a reliable qualitative description of the binding of HSA:NSAIDs. Figure 2 depicts the UV absorption spectra for HSA in the absence and presence of NSAIDs in the molar ratios of 1:0.6, 1:1, and 1:8 at 310 K. The *n* → π* transition in the 260–300 nm region (Figure 2, black lines) occurs due to the presence of aromatic residues in HSA, namely tryptophan (Trp), phenylalanine (Phe), and tyrosine (Tyr) [37]. After adding KTF or KTL to the HSA solution, a significant hyperchromic effect is observed (Figure 2, red lines) with a blue shift occurring in the HSA:KTF (1:8) spectrum, indicating a strong ground-state association [37,46]. To determine whether these shifts occur due to the binding rather than as a consequence of signal addition, the contribution of the non-bound drug was subtracted from the spectrum of the complex (Figure 2, blue lines), resulting in a small hyperchromic effect without a blue shift for HSA:KTF. Thus, there is a weak to moderate ground-state association between HSA and both NSAIDs (HSA:KTF and HSA:KTL) [47].

### 2.2. A Quantitative Evaluation on the Binding of HSA:NSAIDs

**Figure 3:**
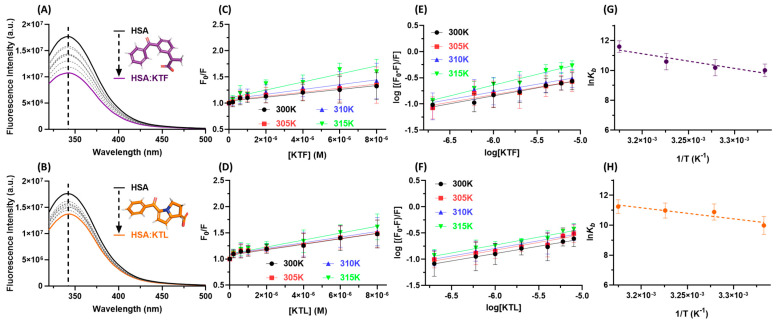
The steady-state fluorescence quenching of HSA by (**A**) KTF and (**B**) KTL at pH 7.4 and 310 K. The Stern–Volmer plots for the interaction of (**C**) HSA:KTF and (**D**) HSA:KTL at eight molar ratios and four temperatures. The double logarithmic plots for the interaction of (**E**) HSA:KTF and (**F**) HSA:KTL. A van’t Hoff plot based on the *K_b_* values for the determination of the thermodynamic parameters of (**G**) HSA:KTF and (**H**) HSA:KTL.

Steady-state fluorescence spectroscopy is one of the most used techniques to determine the quantitative binding parameters of different small compounds with proteins, including NSAIDs binding with albumin [48,49,50]. Figure 3A,B depict the steady-state fluorescence emission of HSA without and upon the successive additions of KTF and KTL, respectively. Since NSAIDs did not cause any shift in the fluorescence spectra, the binding does not perturb the microenvironment around the fluorophores of albumin [51]. The steady-state fluorescence emission of NSAIDs was recorded, and no fluorescence was detected within the region corresponding to the albumin fluorescence emission (320–500 nm range).

The binding parameters obtained using the Stern–Volmer analysis of the steady-state fluorescence data are shown in Figure 3C–H and summarized in Table 1. The Stern–Volmer quenching (*K_SV_*) constant values increased with the increasing temperature, indicating a contribution to the dynamic process. However, the Stern–Volmer plots are linear, and the bimolecular quenching rate (*k_q_*) constant values are approximately three orders of magnitude larger than the maximum diffusion rate constant in water (*k_diff_* ≈ 7.40 × 10^9^ M^−1^ s^−1^ at 298 K, according to the Smoluchowski–Stokes–Einstein theory at 298 K) [52], indicating a ground-state association between HSA and NSAIDs [37], which is in agreement with the UV absorption results.

The number of binding sites (*n*) related to the association of HSA:NSAIDs is close to 1 (Table 1), indicating that the HSA protein interacts with one drug molecule [53]. The binding constant (*K_b_*) values are in the range of 10^4^–10^5^ M^−1^ for HSA:KTF and in the order of 10^4^ M^−1^ for HSA:KTL, indicating moderate binding affinity [27,28,29,30,31,32]. The thermodynamics parameters of the HSA:KTF and HSA:KTL interaction were also determined, and the results are summarized in Table 1. Positive values were observed for both the enthalpy (Δ*H*°) and entropy changes (Δ*S*°), corresponding to an endothermic and entropy-driven binding process controlled by hydrogen bonding and hydrophobic interactions [54]. The negative Gibbs free energy (Δ*G*°) values are compatible with the spontaneity of the binding.

### 2.3. Proofing the Main Fluorescence Quenching Mechanism of HSA Induced by NSAIDs

**Figure 4:**
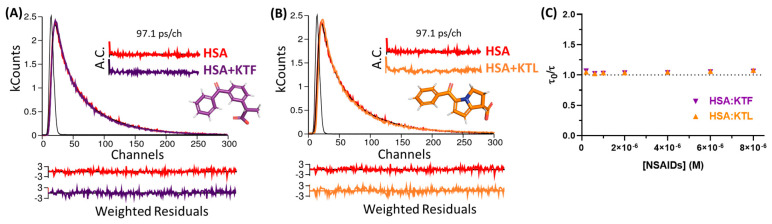
The fluorescence decays measured for HSA (1.0 × 10^−6^ M) both in the absence and presence of (**A**) KTF and (**B**) KTL at the maximum NSAID concentration used in the steady-state fluorescence analysis (8.0 × 10^−6^ M) at room temperature. For a better judgment of the quality of the fit, the weighted residuals (W.R.) and the autocorrelation function (A.C.) are also presented. The black decay curves correspond to the instrumental response function (IRF). (**C**) Stern–Volmer plots based on time-resolved fluorescence data for HSA:KTF and HSA:KTL at 296 K.

Since the steady-state fluorescence analysis detected a possible contribution of a dynamic process in the steady-state fluorescence quenching of albumin, time-resolved fluorescence decays were collected with and without NSAIDs in the same drug concentration used in Section 2.2. The shape of the fluorescence decays of HSA (ranging from 0.20 to 8.0 × 10^−6^ M) exhibited similar profiles. Hence, Figure 4A,B depict the decays of HSA with the highest drug concentration studied (8.0 × 10^−6^ M). Table 2 summarizes the shorter and longer fluorescence lifetimes (τ_1_ and τ_2_, respectively), average lifetime (τ_average_), pre-exponential factors (A_1_ and A_2_), and relative contribution (%Rel). The non-bound HSA has two fluorescence lifetimes in PBS, e.g., τ_1_ = 1.56 ns (18%) and τ_2_ = 5.92 ns (82%), agreeing with the literature [55,56,57], while the HSA:KTF and HSA:KTL complexes decreased by maximums of 0.31 ns (5.8%) and 0.38 ns (7.2%), respectively.

The Stern–Volmer plots of the fluorescence intensities (F_0_/F, Figure 3C,D) show a linear dependence with the drug concentration; however, the dependence of the τ_0_/τ ratio (shown in Figure 4C) is approximately 1 in the range of drug concentrations studied (see also Table 2). This indicates that the contribution of the static quenching process is higher than that of the dynamic phenomenon [37]. Thus, the *K_SV_* values can also be used to estimate the binding constant of the nonfluorescent ground state complex formed between HSA and the drugs under study [37,58]. The *K_SV_* values are in the same order of magnitude compared with *K_b_* values, reinforcing the moderate binding affinity.

### 2.4. Conformational Perturbation of HSA upon NSAID Binding

**Figure 5:**
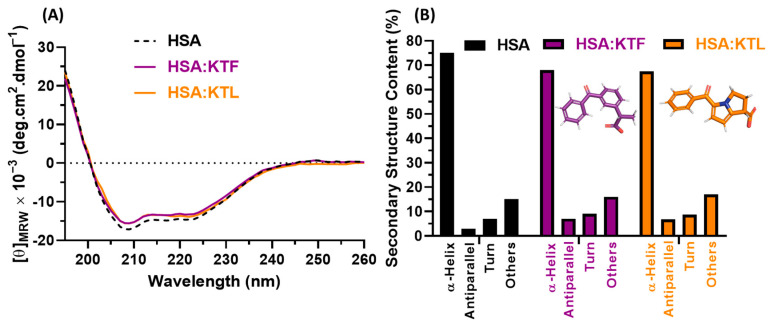
(**A**) Far-UV CD spectra for human albumin (1.0 × 10^−6^ M) without and after the addition of KTF or KTL (8.0 × 10^−6^ M) in PBS at 310 K. (**B**) Corresponding secondary structure content for HSA and HSA:NSAIDs.

Circular dichroism (CD) in the far-UV region is a versatile and simple method to evaluate the perturbation on the secondary structure of a protein upon drug binding [59,60]. Therefore, Figure 5A depicts the far-UV CD spectra for HSA and HSA:NSAIDs in PBS at 310 K. The CD spectra show two minimum peaks, one at 208 nm (π → π* transition) and the other at 222 nm (n → π* transition), which are characteristic of the α-helix secondary structure content [61,62]. The presence of NSAIDs in the albumin/drugs molar ratio of 1:8 results in spectra that are similar in shape and peak position compared with the spectrum of non-bound HSA. The secondary structure contents (%) of non-bound albumin and HSA:NSAIDs are depicted in Figure 5B and summarized in Table 3, showing a slight decrease in the α-helix content (7.1% and 7.6% in the presence of KTF and KTL, respectively), resulting in slight increases in the antiparallel β-sheet, turns, and other structural contents [63]. It was reported that non-bound albumin comprises around 67% α-helices, 10% turns, and 23% random coils [64]. The obtained α-helices content (Table 3) is around 8% higher than that reported in the literature, probably due to the lack of a far-UV CD peak at 190 nm, which is important for an appropriate α-helix calculation (in our case, limitations from the UV-cutoff of PBS, concentration of HSA, and the pathlength of the cell) [65]. These results are consistent with the thermodynamic signature determined for the interaction of HSA with NSAIDs. The conformational rearrangement of HSA after binding to NSAIDs may explain the positive entropic contribution observed for these types of drugs.

### 2.5. An Atomic Point of View of the Interaction of HSA:NSAIDs

**Figure 6:**
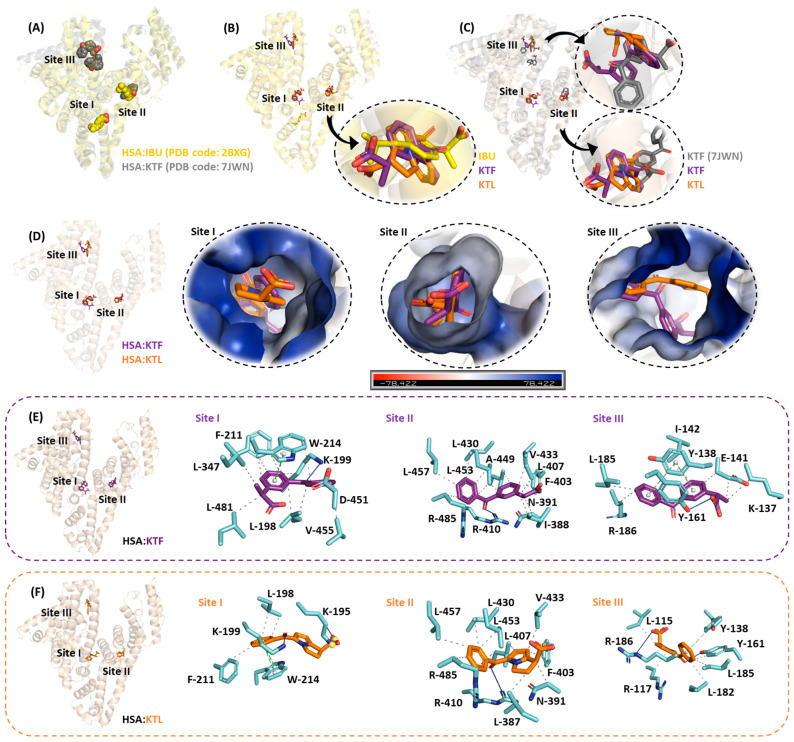
(**A**) The superposition of the X-ray data for HSA:IBU (PDB code: 2BXG) and HSA:KTF (PDB code: 7JWN). The superposition of the best docking pose for HSA:KTF/KTL with the X-ray data for (**B**) HSA:IBU and (**C**) HSA:KTF. (**D**) A cartoon and electrostatic potential map of albumin in the presence of KTF and KTL docked into subdomains IIA, IIIA, and IB. The main amino acid residues and intermolecular forces responsible for the interactions of (**E**) HSA:KTF and (**F**) HSA:KTL in the three main binding sites. The meaning of the color for each compound is indicated in the figure. Hydrogen atoms were omitted for better interpretation, while oxygen and nitrogen are shown in red and dark blue, respectively. The blue lines, green lines, and black dots are indicative of hydrogen bonds, π-stacking, and hydrophobic interactions, respectively.

The HSA structure has three main binding sites, namely sites I, II, and III, which are located in subdomains IIA, IIIA, and IB, respectively [66,67]. To suggest the main binding pose and offer an atomic point of view of the interaction between HSA and NSAIDs, molecular docking calculations were carried out for the three main binding sites. The obtained docking score values (dimensionless) for HSA:KTF were 61.4, 71.2, and 66.0 in sites I, II, and III, respectively, while the corresponding docking score values for HSA:KTL were 55.7, 72.3, and 62.2. Since the highest docking score value was obtained for site II, subdomain IIIA was suggested as the main binding site for the assayed NSAIDs. Figure 6A–C depict the superposition of the docked pose of KTF and KTL using the X-ray data for HSA:IBU [23] and HSA:KTF [33], which indicates the reliability of the in silico trend. The superposition of HSA:KTF and HSA:KTL with the corresponding electrostatic potential map for albumin is shown in Figure 6D. Finally, the intermolecular forces responsible for the stability of the interaction of albumin with NSAIDs are represented in Figure 6E,F, suggesting hydrogen bonding and hydrophobic interactions as the main intermolecular forces responsible for the stability of the complex, agreeing with the experimental thermodynamic parameters [68].

## 3. Discussion

NSAIDs are among the most used medications and are prescribed alone or in conjunction with several other medications. This therapeutic class has applications in managing many inflammatory diseases, both in their chronic and acute manifestations, or in treating other diseases that have inflammatory processes associated with the main conditions, as observed in chronic degenerative diseases [69]. These drugs bind with HSA to be distributed in the bloodstream, particularly impacting the desired therapeutic effects (pharmacokinetic and pharmacodynamic effects) [7]. The biophysical characterization of the interaction between albumin and the NSAIDs, more specifically KTF and KTL, was primarily determined by other authors via spectroscopy without considering some pitfalls, e.g., the inner filter correction and the use of different mathematical approximations without considering the fluorescence quenching mechanisms [28,29,30,31]. Thus, we revised and updated the biophysical characterizations of HSA:KTF and HSA:KTL via multiple spectroscopic techniques combined with molecular docking.

The preliminary binding evaluation was carried out by both ^1^H STD-NMR and UV absorption, demonstrating that NSAIDs are buried into the albumin’s pocket and might interact via ground-state association. Particularly, based on the relative intensity of the ^1^H STD-NMR spectra for HSA:KTL and HSA:KTF, it is observed that aromatic groups interact preferentially with HSA in relation to the aliphatic moiety, supporting the in silico results obtained into subdomain IIIA (site II). Additionally, the ^1^H STD-NMR spectra agree with the reported X-ray data for HSA:KTF that also show an interaction between all of the chemical aromatic moieties of KTF into the pocket of the protein [33]. Meanwhile, Zhu and coauthors [29] observed a strong blue shift of the albumin signal (247–300 nm region) upon the addition of KTF via UV absorption, claiming that there is a strong association between albumin and KTF, and when the drug is inserted into the cavities of the protein, it disrupts the original structure of the biomacromolecule, leading to an unfolding process. Unfortunately, the contribution of the non-bound drug was not subtracted from the complex spectrum, and the authors obtained a fake trend due to the signal addition. After subtraction, our UV absorption data showed a weak hyperchromic effect without any blue or red shift, indicating that KTF does not interact with albumin in a strong way and does not lead to an unfolding process. Our new statement was reinforced by far-UV CD measurements, which detected a slight decrease in the α-helix content (7.1–7.6% range) in the albumin:NSAID molar ratio of 1:8.

The binding of HSA with NSAIDs decreases the steady-state fluorescence of albumin without causing any shift in the maximum fluorescence peak, indicating that KTF and KTL do not perturb the microenvironment around the main fluorophores (Trp, Tyr, and Phe). The same trend was previously reported for albumin with KTF [28,29]; however, Deeps and coauthors [31] reported the opposite trend for KTL (red shift) without exploring the reasons for observing this phenomenon. It is possible that the authors observed a red shift due to the presence of organic solvent in the stock solution of KTL and not due to the binding step. Additionally, we obtained the opposite trend compared with the reported trend of the binding of albumin:IBU (a slightly blue shift, indicating that the binding occurs together with an increase in the hydrophobicity of the microenvironment surrounding the fluorophores [32]). This suggests that even though IBU, KTF, and KTL belong to the same class of NSAIDs, slight differences in their chemical structures might induce some specificities in the binding to albumin.

The ground-state association for HSA:KTF and HSA:KTL previously detected by our UV absorption analysis was reinforced by both shapes of the Stern–Volmer plots and the *k_q_* values combined with the time-resolved fluorescence data. It is important to highlight that the *K_SV_* values indicated a contribution of dynamic fluorescence quenching; however, due to the slight change in the τ_average_ value for albumin in the presence of KTF or KTL, static quenching was predominant. Thus, both *K_SV_* and *K_b_* values can be used to estimate the binding affinity for HSA:NSAIDs [37,58,66,70] as they demonstrate the same order of magnitude and trend. In this case, there are contradictions for the previously reported data to HSA:KTF [28,29]. Bi and coauthors [28] indicated a purely static quenching mechanism (*K_SV_* in the range of 2.97–2.56 × 10^4^ M^−1^) without applying the inner filter corrections and they did not provide any additional analysis to prove this statement. On the other hand, Zhu and coauthors [29] reported a combination of static and dynamic quenching (*K_SV_* 2.9–3.5 × 10^4^ M^−1^), also not considering the pitfalls raised above. Finally, a dynamic quenching mechanism (*K_SV_* 6.77–8.47 × 10^5^ M^−1^) [31] was also previously reported for HAS:KTL without any additional confirmation and/or corrections in the steady-state fluorescence data. In this sense, it is difficult to compare our data with those previously reported in the literature; however, UV absorption, steady-state, and time-resolved fluorescence data clearly indicate a static quenching mechanism as the predominant fluorescence quenching mechanism of albumin induced by KTF or KTL.

As a drug carrier, HSA may aid in the selective delivery of NSAIDs until the targets are reached and facilitate drug access into the cell via receptor mechanisms (moderate binding affinity for HSA). Since the *K_SV_* and *K_b_* values are in the order of 10^4^ M^−1^ at 310 K, KTF and KTL bind moderately with albumin, which is favorable for achieving the ideal pharmacokinetic profile [70,71]. The obtained *K_SV_* and *K_b_* values for KTF and KTL at human body temperature (310 K) have differences in the range of 0.7- and 3.4-fold between them, which is supported by the experimental pharmacokinetic profile that also indicates that these drugs bind extensively to plasma albumin with a difference in the apparent volume of distribution (*V_d_*, the ratio of the total amount of drugs in the body to the plasma concentration of the drugs) of around 1.6-fold between them [72,73].

In all evaluated temperatures, negative Δ*G*° values were obtained, which are consistent with the spontaneity of the binding of HSA:NSAIDs, and since there are positive values for both Δ*H*° and Δ*S*°, only the entropic value contributes to the negative Δ*G*° value; therefore, the associations of HSA:KTF and HSA:KTL are entropically driven. According to Ross and Subramanian [68], Δ*H*° > 0 and Δ*S*° > 0 are indicative that hydrogen bonding and hydrophobic interactions might contribute significantly to complex stability, agreeing with the in silico data in subdomain IIA. Zhu and coauthors [29] reported quite similar thermodynamic values (Δ*H*° ≈ 74.5 kJmol^−1^ and Δ*S*° ≈ 0.334 kJmol^−1^K^−1^) to albumin:KTF, while Bi and coauthors [28] reported a different thermodynamic trend (Δ*H*° ≈ −22.2 kJmol^−1^ and Δ*S*° ≈ 0.00548 kJmol^−1^K^−1^ at 308 K). This probably occurred due to the differences in the detection of the main fluorescence quenching mechanism by steady-state measurements. Finally, the reported Δ*H*° and Δ*S*° values for albumin:KTL are not reliable because Deepa and coauthors [31] applied the van’t Hoff approximation using only two temperatures.

Overall, the obtained data corrected and updated those previously reported by other authors who did not consider the inner filter corrections, spectra background, and the identification of the leading mathematical approach to determine the binding affinities of HSA:KTF and HSA:KTL. Additionally, the NSAIDs KTF and KTL showed some spectroscopic differences with those reported for albumin:IBU [32,49]; however, their binding constants at human body temperature are in the order of magnitude of 10^4^ M^−1^.

## 4. Materials and Methods

### 4.1. General Materials

All reagents, including KTF, KTL, Ludox^®^, and HSA (purity ≥ 99%, catalog number A3782), were provided by Merck KGaA company (Darmstadt, Germany) and used without further purification. The phosphate-buffered solution (PBS) was prepared with 137 mM sodium chloride (NaCl), 2.7 mM potassium chloride (KCl), 8 mM disodium phosphate (Na_2_HPO_4_), and 2mM potassium dihydrogen phosphate (KH_2_PO_4_) to achieve pH 7.4.

### 4.2. Nuclear Magnetic Resonance (NMR) Measurements

The ^1^H NMR spectra were obtained at 278 K using 600 μL PBS (pH 7.4) and 10% (*v*/*v*) of D_2_O. The measurements were performed on a 400 MHz Bruker Avance III NMR spectrometer (Bruker, MA, USA) equipped with a BBFO 5 mm double resonance broadband direct detection z-gradient probe head. For the saturation transfer difference NMR (^1^H STD-NMR) experiments, a pseudo-2D pulse sequence was utilized, which included spoil pulses to eliminate any residual magnetization during the relaxation delay. Specifically, two trim pulses of 1.5 and 2.5 ms were followed by a 2 ms gradient pulse on the *z*-axis. To selectively saturate HSA (20 × 10^−6^ M), cascades of 50 ms Gaussian-shaped pulses with a field strength of 80 Hz were employed with a 1 ms delay between successive pulses. The total saturation time for the STD measurements was set at 3 s with a recycling delay of 2 s in experiments consisting of 768 scans. Selective saturation of the protein was successfully achieved by establishing the on-resonance frequency at 0.85 ppm. To obtain the reference (off-resonance) spectrum, the irradiation frequency was adjusted to 40 ppm. The concentrations of NSAIDs and human albumin were 400 and 20 × 10^−6^ M, respectively. Bruker Topspin version 3.4 software was employed to process the NMR spectra.

### 4.3. UV Absorption Measurements

The UV absorption spectra were recorded in a SpectraMax iD5 Multi-Mode Microplate Reader (Molecular Devices Corporation Sunnyvale, CA, USA) in the 230–375 nm range at 310 K. Three different spectra for HSA:NSAIDs were obtained using PBS signal as the baseline: non-bound HSA solution (1.0 × 10^−6^ M), NSAID solutions (0.6, 1.0, and 8.0 × 10^−6^ M), and a mixture of HSA:NSAIDs under the same concentrations that were used for the isolated compounds.

### 4.4. Steady-State Fluorescence Measurements

The steady-state fluorescence spectra were recorded in a SpectraMax iD5 Multi-Mode Microplate Reader (Molecular Devices Corporation Sunnyvale, Alviso, CA, USA) with an excitation wavelength (λ_exc_) of 280 nm at 300, 305, 310, and 315 K. The measurements were obtained in the 320–500 nm range using the corresponding background corrections. The concentration of NSAIDs was successively increased to a fixed concentration of albumin (1.0 × 10^−6^ M) until final concentrations of 0.2, 0.6, 1.0, 2.0, 4.0, 6.0, and 8.0 × 10^−6^ M. The inner filter correction was applied following the literature [74,75]. To obtain quantitative parameters of the binding affinity of HSA:NSAIDs, several mathematical approximations were used, namely the Stern–Volmer, double-logarithmic, van’t Hoff, and Gibbs’ free energy [37,76,77,78].

### 4.5. Time-Resolved Fluorescence (TRF) Measurements

Time-resolved fluorescence (TRF) decays were obtained through a home-built time-correlated single photon counting (TCSPC) apparatus that was previously described [79]. The measurements were obtained at room temperature for HSA (1.0 × 10^−6^ M, in PBS) and HSA:NSAIDs (0.00, 0.20, 0.60, 1.0, 2.0, 4.0, 6.0, and 8.0 × 10^−6^ M, in PBS) with excitation at 282 nm using 1024 channels and a 97.1 ps/channel resolution. Alternate measurements of the pulse profile at 282 nm and the sample emission were collected until 2500 counts, at the maximum, were reached. The instrumental response function (IRF) was collected using a Ludox^®^ dispersion. Deconvolution of the fluorescence decay curves was performed using the modulation function method, as implemented by G. Striker in the SAND v1.0 software, as previously reported in the literature [80]. The average fluorescence lifetime (τ_average_) was determined following the previous publication [46].

### 4.6. Circular Dichroism (CD) Measurements

Circular dichroism spectra were recorded on an Olis DSM-20CD spectrophotometer (OLIS, Inc., Bogart, GA, USA) with a Quantum Northwest CD 150 temperature controller system (Quantum Northwest, Inc., Liberty Lake, WA, USA). The far-UV CD spectra (from 195 to 260 nm) for albumin (1.0 × 10^−6^ M, in PBS) were recorded in the absence and presence of the maximum concentration of NSAIDs used in the steady-state fluorescence measurements (8.0 × 10^−6^ M, in PBS) in a 0.1 cm pathlength cuvette under N_2_ atmosphere. The average spectra obtained from three successive runs were corrected by the subtraction of the buffer signal. The results were normalized [47], and the secondary structure content was estimated through an analysis of the far-UV spectra using the online server Beta Structure Selection (BeStSel, http://bestsel.elte.hu/index.php, accessed on 22 January 2024) [81].

### 4.7. Molecular Docking Procedure

The chemical structure for non-bound HSA was obtained from the Protein Data Bank (PDB) with access code 3JRY [82]. The chemical structures for KTF and KTL were built and energy-minimized with the Spartan’14 software (Wavefunction, Inc., Irvine, CA, USA). The molecular docking calculations were performed with the GOLD 2022.3.0 software (Cambridge Crystallographic Data Centre, Cambridge, UK) with an 8 Å radius around subdomains IIA, IIIA, and IB following previous publications [47,77,78]. Figures for the docking poses were generated using PyMOL 3.0 Delano Scientific LLC software (Schrödinger, New York, NY, USA).

## 5. Conclusions

The binding of HSA with NSAIDs decreases the steady-state fluorescence of albumin without causing any shift in the maximum fluorescence peak. The binding of HSA:NSAIDs is spontaneous, endothermic, and entropically driven, leading to a conformational rearrangement of HSA with a slight decrease in the α-helix content (7.1% to 7.6%). Particularly, based on the relative intensity of the ^1^H STD-NMR spectra for HSA:KTL and HSA:KTF, the aromatic groups interact preferentially with HSA in relation to the aliphatic moiety, supporting the in silico results obtained for subdomain IIIA (site II). The *K_SV_* values indicate a contribution of the dynamic fluorescence quenching mechanism; however, due to the slight change in the τ_average_ values, static quenching is predominant. Thus, both the *K_SV_* and *K_b_* values can be used to estimate the binding affinity for HSA:NSAIDs, which are in the same order of magnitude (10^4^ M^−1^) for those reported to IBU at human body temperature.

## Figures and Tables

**Table 1:** The steady-state fluorescence quenching parameters for the interaction of HSA:NSAIDs at four different temperatures in pH 7.4.

System	T (K)	*K_SV_* (×10^4^) (M^−1^)	*k_q_* (×10^12^) ^1^ (M^−1^s^−1^)	*n*	*K_b_* (×10^4^) (M^−1^)	Δ*H*° (kJmol^−1^)	Δ*S*° (kJmol^−1^K^−1^)	Δ*G*° (kJmol^−1^)
HSA:KTF	300	3.68 ± 0.11	7.13 ± 0.08	0.785 ± 0.06	2.21 ± 0.10	81.3 ± 19.9	0.353 ± 0.065	−24.6 ± 0.5
	305	3.90 ± 0.12	7.56 ± 0.10	0.790 ± 0.08	2.64 ± 0.16			−26.4 ± 0.2
	310	4.62 ± 0.12	8.95 ± 0.11	0.802 ± 0.08	3.98 ± 0.13			−28.1 ± 0.2
	315	13.2 ± 1.1	25.6 ± 0.85	0.911 ± 0.05	10.8 ± 0.10			−29.9 ± 0.5
HSA:KTL	300	1.07 ± 0.10	2.07 ± 0.19	0.780 ± 0.08	2.17 ± 0.14	61.4 ± 20.9	0.289 ± 0.068	−25.3 ± 0.5
	305	1.12 ± 0.11	2.17 ± 0.21	0.795 ± 0.06	5.31 ± 0.11			−26.7 ± 0.2
	310	1.35 ± 0.10	2.62 ± 0.19	0.831 ± 0.06	5.85 ± 0.12			−28.2 ± 0.2
	315	1.94 ± 0.09	3.76 ± 0.17	0.893 ± 0.04	7.63 ± 0.11			−29.6 ± 0.5

^1^ Using the τ_average_ obtained in this work for non-bound HSA (5.16 ns).

**Table 2:** Fluorescence lifetimes (τ_1_ and τ_2_), average lifetimes (τ_average_), pre-exponential factors (A_1_ and A_2_), and relative contributions (%Rel) for HSA, HSA:KTF, and HSA:KTL.

System	[NSAIDs] × 10^−6^ (M)	τ_1_ (ns)	τ_2_ (ns)	A_1_	A_2_	%Rel (τ_1_)	%Rel (τ_2_)	τ_average_ (ns)	τ_0_/τ_average_
HSA:KTF	0.00	1.56	5.92	0.448	0.552	18	82	5.15	------
	0.20	1.43	5.75	0.517	0.483	21	79	4.84	1.07
	0.60	1.41	5.72	0.429	0.571	16	84	5.05	1.02
	1.00	1.40	5.73	0.444	0.556	16	84	5.02	1.03
	2.00	1.50	5.77	0.461	0.539	18	82	4.99	1.03
	4.00	1.48	5.74	0.460	0.540	18	82	4.97	1.04
	6.00	1.51	5.69	0.467	0.533	19	81	4.90	1.05
	8.00	1.55	5.67	0.471	0.529	20	80	4.86	1.06
HSA:KTL	0.00	1.55	5.89	0.431	0.569	17	83	5.17	------
	0.20	1.58	5.88	0.568	0.532	22	78	4.92	1.05
	0.60	1.52	5.86	0.471	0.529	9	81	5.05	1.02
	1.00	1.52	5.81	0.482	0.518	20	80	4.97	1.04
	2.00	1.53	5.80	0.480	0.520	20	80	4.96	1.04
	4.00	1.55	5.80	0.494	0.506	21	79	4.92	1.05
	6.00	1.47	5.75	0.506	0.494	21	79	4.86	1.06
	8.00	1.47	5.71	0.518	0.482	22	78	4.79	1.08

**Table 3:** Structural contents (%) for non-bound HSA, HSA:KTF, and HSA:KTL in albumin/drug molar ratio of 1:8.

Sample	α-Helix	β-Sheets (Antiparallel)	Turn	Others ^1^
HSA	75.1	2.9	6.9	15.1
HSA:KTF	68.0	7.0	9.0	16.0
HSA:KTL	67.5	6.8	8.7	17.0

^1^ Includes random coil.

## Data Availability

All data are contained within the article.
